# Effects of postural change and mobilization on hemoglobin levels in ICU patients: a prospective intervention study

**DOI:** 10.1186/s40635-026-00961-x

**Published:** 2026-08-24

**Authors:** Tasja Slijkerman, Huub L. A. van den Oever, Kim Kamphorst, Jacques B. de Kok, Alfred L. van Steveninck

**Affiliations:** Deventer Hospital, Deventer, The Netherlands

**Keywords:** Hemoglobin, Anemia, Transfusion, Posture, Mobilization, ICU

## Abstract

**Background:**

In the absence of overt bleeding, a gradual decline in hemoglobin (Hb) levels is common following admission of patients to the Intensive Care (ICU). While effects of posture on measured Hb levels have been reported, knowledge about their occurrence and relevance in ICU patients is largely lacking. Such postural Hb changes are generally considered to result from plasma volume shifts. We studied the effects of passive and active postural changes on measured Hb values in hemodynamically stable, non-bleeding ICU patients.

**Methods:**

In a single-center uncontrolled interventional study in hemodynamically stable adult ICU patients without evidence of bleeding, arterial Hb was measured after ≥ 6 h of supine bedrest, 30 min after a passive change to sitting in bed and subsequently after mobilizing patients to a chair. Baseline values for erythrocyte sedimentation rate (ESR) and C-reactive protein (CRP) and changes in serum albumin were recorded to explore potential correlations with Hb changes. The data was analyzed using linear mixed modelling.

**Results:**

Twenty-three patients (median age 72 years, 57% male) were included in the study. The average baseline Hb obtained from the mixed model analysis was 11.1 g/dL (95% confidence interval (CI): 10.0–12.1 g/dL ). A passive change to a sitting position in bed resulted in an increase of 0.4 g/dL (95% CI: 0.2–0.5, *p* < 0.001), with a further increase of 0.5 g/dL (CI: 0.3–0.6, *p* < 0.001) after mobilization to a chair. There was a positive correlation between changes in Hb and albumin (supine to sitting in bed: Spearman ρ = 0.76, *p* < 0.001, supine to mobilized in a chair: Spearman ρ = 0.72, *p* < 0.001). No significant correlations were observed for Hb changes and ESR, or CRP.

**Conclusions:**

In ICU patients, modest but significant increases in Hb values were demonstrated following a change from supine to a sitting position, with further increases upon mobilization to a chair. The observed postural Hb changes could affect transfusion decisions for Hb values near generally accepted transfusion thresholds.

## Background

Significant declines in hemoglobin (Hb) in the absence of overt bleeding are common within days following ICU admission, with a continuing decline beyond the third day occurring mostly in patients with sepsis and sepsis-related organ failure [1]. Blood transfusions are frequent in ICU patients, with Hb values and clinical assessment guiding transfusion decisions. However, blood transfusions have been associated with impaired outcome [2, 3, 4], prompting strategies to limit iatrogenic blood loss and stimulate erythropoiesis [5, 6, 7] in ICU patients.

A reduced circulatory life span and diminished red blood cell production are generally cited as causes for anemia in ICU patients [8]. These are multifactorial, with occult bleeding, diagnostic sampling, hemolysis and eryptosis contributing to a reduced circulatory life span, while elemental/nutritional deficiencies and bone marrow suppression by inflammatory mediators are cited as causes for diminished erythropoiesis. Hemodilution resulting from fluid resuscitation is often cited as a potential cause for initial Hb declines, mostly with rapid administration of fluids [9]. However, its overall contribution to Hb declines in ICU patients appears to be limited [1, 10]. Although postural effects on hemoglobin measures have been documented, they are not cited in reviews on anemia in ICU patients [1, 3, 4, 8].

Postural variations in hemoglobin have been attributed to intravascular volume shifts, which were first demonstrated in a dye dilution study in 1928 [11]. Over time, more sophisticated analyses have confirmed the posture dependency of total plasma volume, with increased hydrostatic pressures causing a fluid shift to the interstitium. Reductions in plasma volume ranging from 5.1% [12] to 16.2% [13] have been reported upon standing but were less pronounced with a shift from supine to a sitting [12, 14]. Likewise, increases in Hb ranging from 5.5% to 10.8% have been reported with standing [13, 15, 14], while an increase of 2.3% was demonstrated following a shift from supine to sitting [14]. A decline of serum albumin concentrations following prolonged bed rest has equally been associated with plasma volume shifts [16], although serum albumin changes did not correspond to acute plasma volume shifts in an experimental setting [17]. Still, serum albumin is widely associated with intravascular volume through its contribution to colloid oncotic pressure.

Thus far, two studies have addressed postural effects on measured Hb in patients. A median increase in venous Hb of 5.2% has been published for hospitalized patients, following mobilization to a chair after six hours of recumbency [18]. Recently, a 6.7% icrease in venous Hb was also reported for hemodynamically stable ICU patients following a change from supine to a more upright position in bed [19].

The current study aimed to evaluate the effects of posture on arterial Hb values in hemodynamically stable, spontaneously breathing, non-bleeding ICU patients. Both passive and active mobilization were included to explore potential effects of physical activity in addition to postural changes alone. Correlations with concomitant changes in serum albumin were evaluated as a potential indicator of changes in plasma volume. Correlations with measures of CRP and ESR were included as increased erythrocyte aggregation in inflammatory states may induce intravascular sedimentation of erythrocytes [20]. This could be of interest when mechanisms other than a plasma volume shift are involved in postural Hb changes.

## Methods

A prospective, interventional study was conducted on a 12-bed general ICU at the Deventer Hospital in the Netherlands. The study was registered (clinicaltrials.gov NL86966.100.24), approved by the.

Medical Research Ethics Committee (MEC-U, R24.044), and conducted in accordance with the World Medical Association Declaration of Helsinki, 2013 revision. Informed consent was obtained from all participants in the study which started in August 2024 and was completed in January 2025.

## Patients

ICU patients aged ≥ 18 years, were eligible if they had an arterial catheter and were able to remain seated for 30 min after mobilization to a chair. Exclusion criteria included artificial ventilation, treatment with noradrenaline > 0.05 µg/kg/min or argipressin, active bleeding, blood transfusions or >3 L of intravenous (IV) fluids within 24 h before measurements, > 500 ml of IV fluids within 4 h of measurements, diuretic treatment, decompensated right heart failure, pulmonary hypertension, pulmonary embolism and hematological disorders.

## Study procedure

**Fig. 1:**
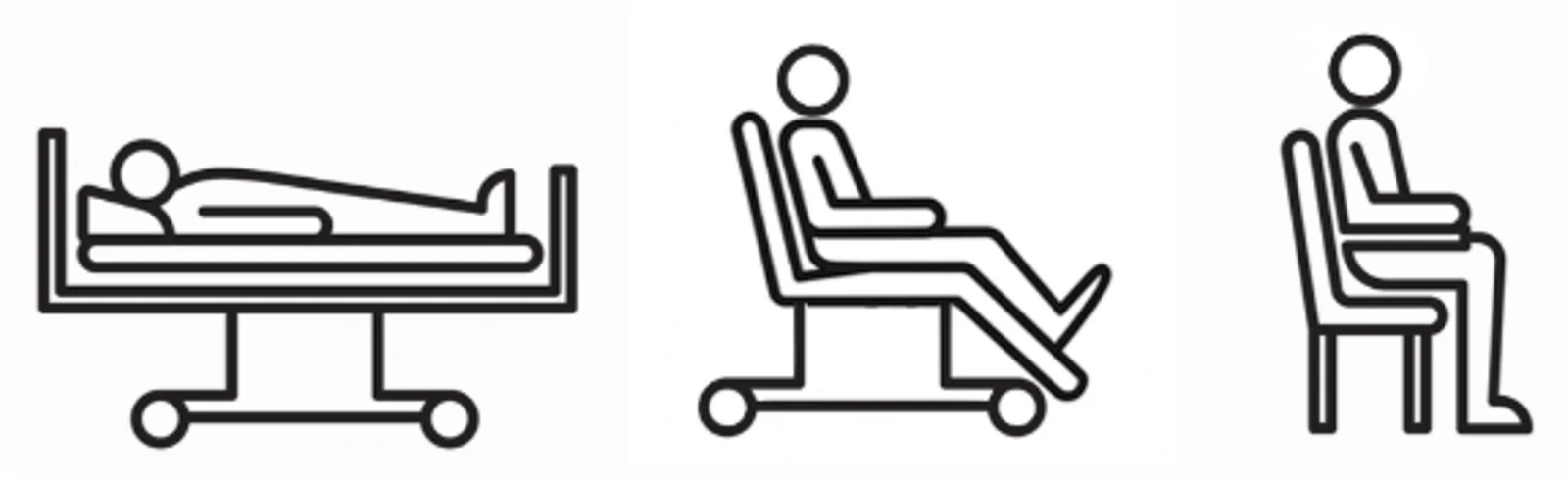
Patient positions for consecutive Hb measurements

Blood samples for Hb, albumin, ESR, and CRP were taken from an indwelling arterial catheter at 6:00 a.m., after patients had been supine for ≥ 6 h. Subsequently, patients were brought to a sitting position in bed (≥ 70-degree head-rest elevation and lower legs dependent; Fig. 1), with a second sample for Hb and albumin taken 30 min after assuming this position. Following daily nursing care, patients were then mobilized into a chair with a third sample for Hb and albumin taken after 30 min. Sample times were not fixed, but the sequence of positional changes preceding blood sampling was equal for all participants. For patients remaining in the ICU, a fourth sample (Hb and albumin) was taken the next morning following ≥ 6 h of recumbency.

## Data management and statistics

A sample size calculation was based on a 2.7% biological variation of Hb and a 4% technical variability of laboratory measurements, with a combined standard deviation of 2.41. The effect size was deemed clinically relevant if it exceeded biological variation. To achieve a power of 80% and a significance level of 5% (two-sided), 23 participants would need to be included with an assumed correlation of 0.6 between measurements.

Continuous variables were assessed for normality using visual inspection of histograms and Q–Q plots. Normally distributed data are presented as mean ± standard deviation (SD), whereas non-normally distributed data are reported as median with interquartile range (IQR). Differences in hemoglobin concentrations across positions (supine, sitting, chair) were analyzed using a linear mixed-effects model with patient position as a categorical fixed effect and patient ID as random intercept to account for repeated measurements. The mixed-effects model accounted for baseline differences in Hb between patients when estimating the average effect of posture. A model allowing the magnitude of the posture effect to vary between patients (random slope) was explored, but this model did not converge and could therefore not be reliably interpreted. Consequently, results are based on the model assuming a common effect of posture across patients, while accounting for differences in individual baseline Hb values.

Secondary endpoints included correlations of Hb changes with changes in albumin and correlations of total Hb change and baseline values for CRP and ESR. Correlations were analysed using Spearman’s correlation coefficients given a non-normal distribution of the data. P-values < 0.05 were considered statistically significant. Statistical analyses were performed using SPSS version 29.

## Results

Twenty-three patients were included in the study, with all patients adhering to the study protocol. Patient characteristics are shown in Table 1.

**Table 1:** Patient characteristics

Patient characteristics	*N* = 23
Age	72 (60–77) years
Gender	13 male, 10 female
Weight	76.4 ± 14.0 kg
Height	173.9 (± 9.5) cm
Body Mass Index (BMI)	25.3 (± 4.2) kg/m^2^
Days in ICU	4 (2–5)
Diagnosis at admission	
• Sepsis/infection	9 (39%)
• Respiratory failure	6 (26%)
• Postoperative care	6 (26%)
• Other	2 (9%)
Apache-IV score	65 (51–84)
SAPS-II score	38 (25–46)
Hb at hospital admission*	12.4 (11,1–16,0 ) g/dL
Hb at ICU admission*	11.9 (10.5–13.2 ) g/dL
Hb preceding postural changes#	10.8 (9.3–12.7) g/dL

N (%); Median (IQR); Mean (±SD). * Conditions with respect to posture and recent activity at the time of blood sampling not registered. #) Blood sampled after > 6 hrs of recumbency

**Fig. 2:**
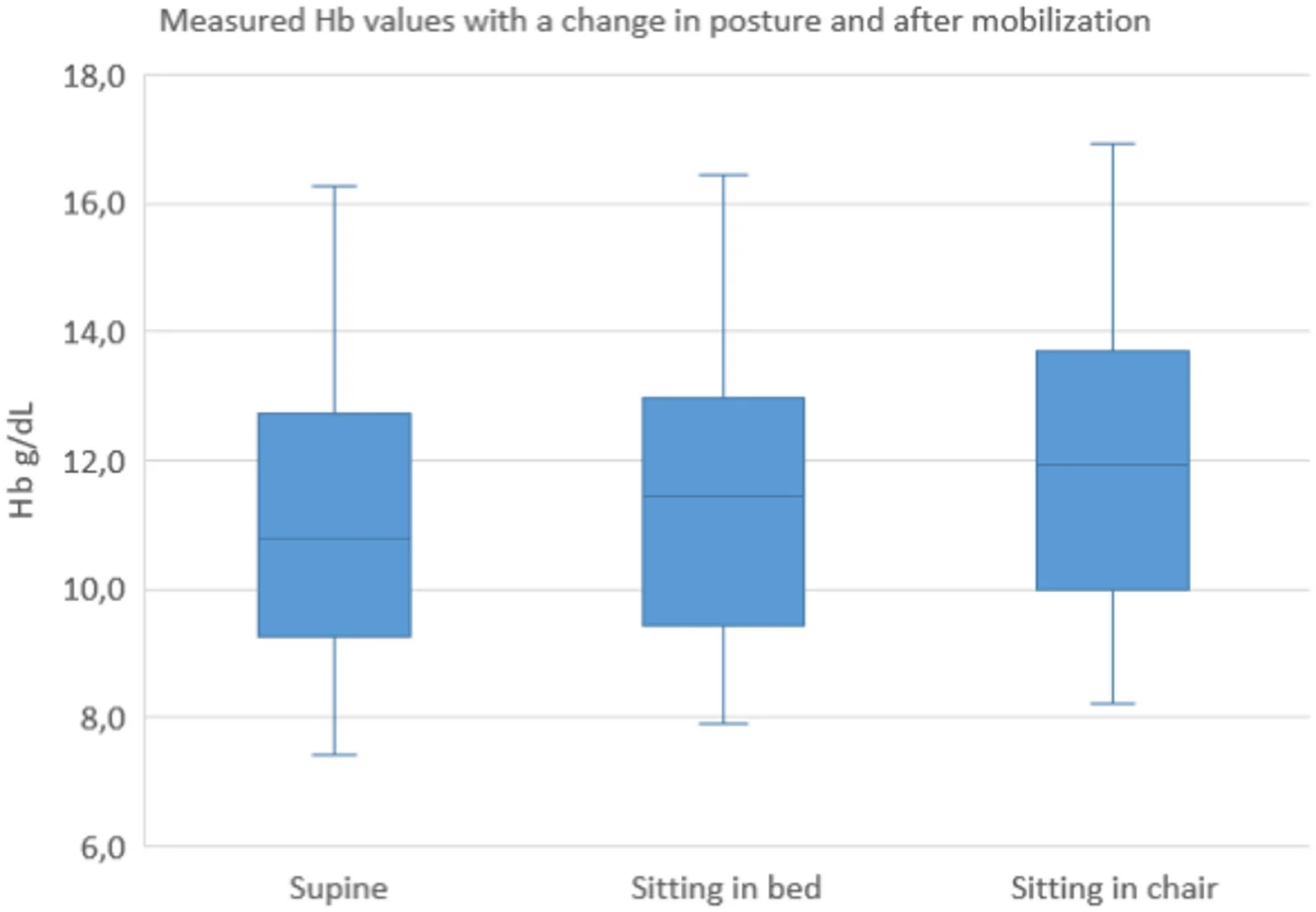
Box plot indicating the median, interquartile range and 1.5 x IQR spread for measured Hb values (g/dL ) in 23 patients. Median Hb values increased from 10.8 g/dL at baseline to 11.4 g/dL with patients sitting in bed and to 11.9 g/dL following mobilization to a chair

**Fig. 3:**
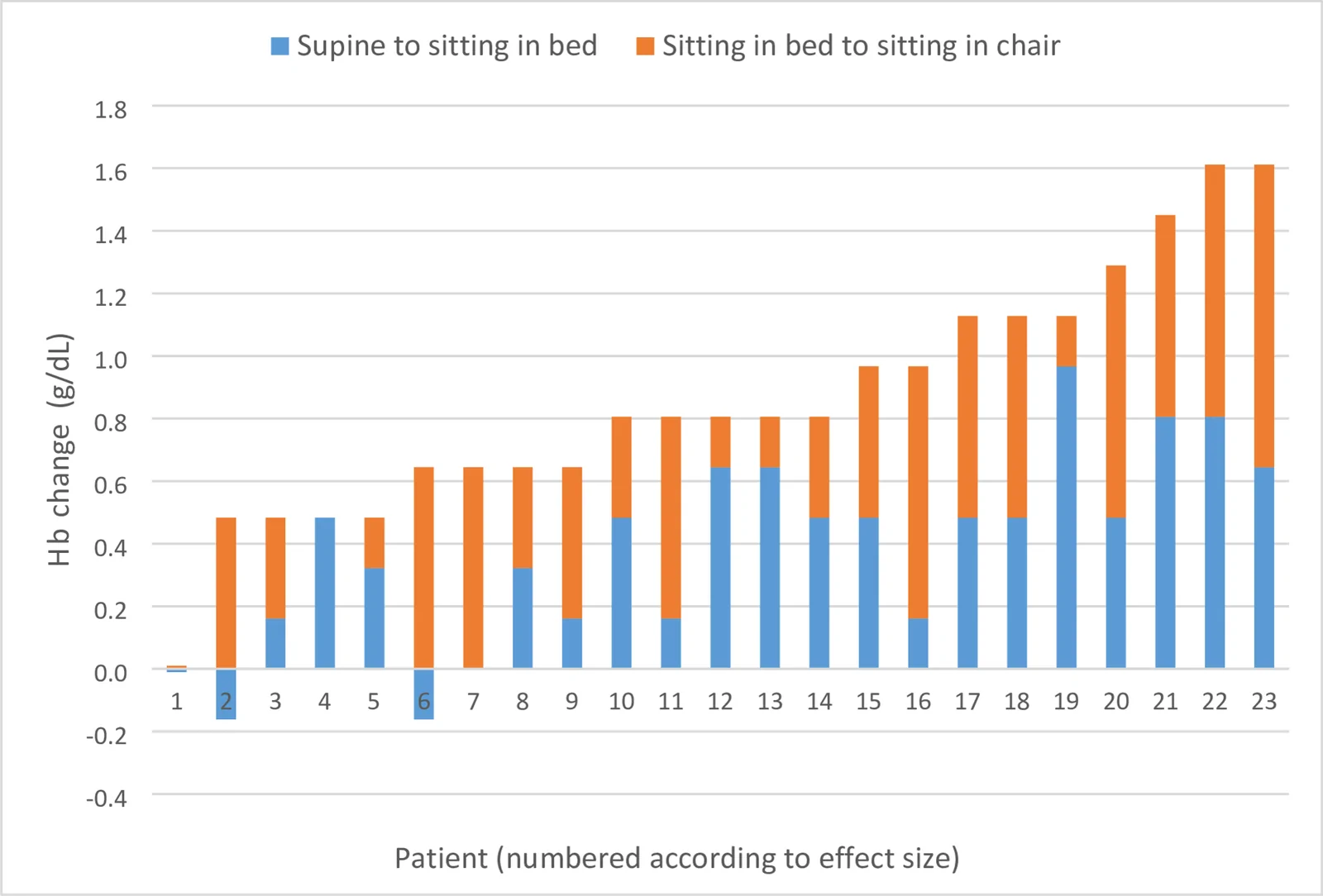
Hb changes (g/dL) for individual patients arranged according to their total effect size

A boxplot of measured Hb values for each study condition is shown in Fig. 2, indicating a 5.6% increase in median values after the passive change to sitting in bed and a total 10.2% increase in median values following mobilization to a chair. Compared to baseline, individual Hb changes ranged from − 0.2 g/dL to + 1.0 g/dL following a passive change to a seated position and from 0.0 g/dL to + 1.6 g/dL after subsequent mobilization to a chair. A graphical representation of changes in individual patients is shown in Fig. 3.

The mixed model analysis resulted in an estimated mean Hb of 11.07 g/dL at baseline, with a 3.5% increase to 11.46 g/dL following the passive postural change to sitting in bed, and an additional 4.3% increase to 11.93 g/dL after mobilization to a chair (Table 2).

**Table 2:** Estimates of fixed effects for the effect of posture on Hb levels. Supine posture was used as the reference category

Fixed effect	Estimate (β) (mmol/L)	95% CI	*p*-value
Intercept (Supine)	11.07	10.04–12.12	
Supine to sitting in bed	0.39	0.24–0.53	< 0.001
Lying in bed to sitting in chair	0.47	0.32–0.60	< 0.001
Supine to sitting in chair	0.85	0.71–0.98	< 0.001

CI = confidence interval

A fourth Hb measurement in supine position, obtained 24 h after baseline, was available for a subset of eight patients. Secondary analysis did not reveal a significant change in Hb compared to baseline (mean difference: -0.21 g/dL, 95% CI: -0.47–0.05, *p* = 0.1).

The median measured albumin concentration at baseline was 25.5 g/L (IQR: 22.7–30.4 g/L). Compared to baseline, albumin concentrations increased by 1.6% to 25.9 g/L (IQR: 24.0–30.9 g/L) after a passive change to sitting in bed and by 5.9% to 27.0 g/L (IQR: 25.4–32.0 g/L) following mobilization to a chair. There was a positive correlation between Hb changes and changes in albumin (supine to sitting in bed: Spearman ρ = 0.76, *p* < 0.001, supine to mobilized in a chair: Spearman ρ = 0.72, *p* < 0.001).

At baseline, ESR and CRP were generally elevated, with a median ESR of 30 mm/h (IQR: 13 − 54) and a median CRP of 61 mg/L (IQR: 13 − 146). No significant correlations were found between ESR or CRP and the total change in Hb across postural changes, with Spearman’s correlation coefficients of -0.21 (*p* = 0.34) for ESR and − 0.10 (*p* = 0.66) for CRP.

## Discussion

This study aimed to determine whether posture and mobilization affect arterial Hb concentrations in hemodynamically stable, non-bleeding ICU patients. The data reveal a near universal increase in Hb values after a passive change from a supine position to sitting in bed, with a further increase following mobilization to a chair. While not large, the Hb changes observed in this study will be clinically relevant at Hb-levels near established transfusion triggers.

Our findings align with previous reports of postural effects in healthy volunteers [13, 14, 15] and non-ICU patients [18], as well as those in a recent study in ICU patients [19]. The Hb changes may be explained by the prevailing hypothesis of plasma volume shifts causing hemoconcentration [13, 21, 22]. However, at an estimated 7.8% (modelled) to 10.2% (measure) increase in Hb folowing the combined interventions, the measured effect far exceeded the 1.5% − 3.4% change in plasma volume between supine and seating positions that has been reported for healthy subjects [12, 14]. An increased capillary leak in patients, relative to that in younger healthy subjects, may be suggested to explain this difference. The smaller increase in serum albumin as compared to Hb values could support this, as capillary leak results in protein extravasation [23] while convective albumin losses [17] increase at elevated hydrostatic pressures [24]. However, with 60–70% of total albumin residing in the extracellular space [25], lymphatic return also has a significant impact on serum albumin concentrations. As lymphatic return increases with an upright posture [26] muscular activity and increased spontaneous breathing [27], the interpretation of increases in serum albumin and the demonstrated correlation with Hb changes remains complex.

The large Hb increases relative to previously reported plasma volume shifts, may also point at other factors involved in postural Hb changes. For example, circulatory changes could affect Hb measures in case of an inhomogeneous distribution of erythrocytes within the vascular system. Such inhomogeneities are known to occur in the microcirculation [28, 29] and in larger vessels [30, 31, 32]. Of interest, gravity-oriented erythrocyte sedimentation has been demonstrated in human venules and veins; by microscopy as early as in 1958 [33] and with radiological examinations in later studies [34, 35, 36]. As erythrocyte sedimentation has been associated with low flow conditions [37, 38], a link between Hb changes and circulatory effects induced by mobilization is conceivable. In our study, a contribution of inflammatory states to Hb changes was not demonstrated. However, ESR and CRP levels were only slightly elevated in our patients, limiting the potential to detect a correlation with Hb changes. Further studies will be needed to substantiate a hypothesis of erythrocyte sedimentation affecting Hb measures in ICU patients.

While the observed Hb changes in our study may seem small on average, they appear to be common and are of concern for patients with Hb values near accepted transfusion triggers. Transfusion guidelines indicate that red blood cell transfusions are to be *considered* for patients with Hb values below threshold values. However, as clear evidence for benefits of red blood cell transfusions is lacking for most patients [39] except those with acute coronary syndromes [40] or acute brain injury [41], a substantiated decision is not easily made. The fact that Hb levels may show a similar increase upon a change in posture as compared to the transfusion of a unit of red blood cells underscores the limited support for such decisions without evidence of a clinically relevant deficit in oxygen transport.

In our study, postural effects were measured on average four days after admission to the ICU. Larger Hb declines have been reported in the first three days following ICU admission [1, 4, 10] and other factors contributing to anemia may prevail in this early phase. Compared to a recent study in ICU patients [19], Hb declines in our study were smaller following a similar passive postural change but slightly larger after mobilization to a chair. In that study blood samples were taken one day earlier on average and from venous blood, while patient characteristics also differed. These factors, along with the small sample sizes, may have influenced the observed effects. Still, both studies clearly add postural anemia to the differential diagnosis of Hb declines in ICU patients.

## Conclusion

This study demonstrates an effect of posture and mobilization on measured Hb values in ICU patients. These findings may have implications for the interpretation of Hb measurements in the ICU. However, as protocol requirements limited us to the study of stabilized and spontaneously breathing patients, results may not apply to all ICU patients. While postural Hb changes are generally assumed to result from plasma volume shifts, results suggest that the effects of active mobilization on measured Hb may not be caused by posture-induced plasma volume-shifts alone.

## Data Availability

The datasets used and/or analysed during the current study are available from the corresponding author on reasonable request.

## Abbreviations

BMI: Body Mass Index
CI: Confidence Interval
CRP: C-Reactive Proteïn
ESR: Erythrocyte Sedimentation Rate
Hb: Hemoglobin
ICU: Intensive Care Unit
IQR: Inter Quartile Range
SD: Standard Deviation
